# Loving‐Kindness Meditation: Systematic Review of Neuroimaging Correlates in Long‐Term Practitioners and Clinical Implications

**DOI:** 10.1002/brb3.70372

**Published:** 2025-02-28

**Authors:** Kiren Bashir, Stephen B. Edstrom, Sara J. Barlow, Danielle Gainer, Jeffrey D. Lewis

**Affiliations:** ^1^ Wright State University Boonshoft School of Medicine Dayton Ohio USA; ^2^ Wright Patterson Medical Center Wright‐Patterson Air Force Base Ohio USA

**Keywords:** loving‐kindness meditation, mental health, neuroplasticity

## Abstract

**Introduction:**

Loving‐kindness meditation (LKM), a meditation type focused on nurturing love and compassion for oneself and others, has been shown to provide mental health benefits, and LKM interventions are being investigated for mental disorders. The benefits of long‐term practice, such as increased self‐compassion, greater cognitive and affective empathy, and prosocial behavior, are proposed to be due to neuroplastic changes that support well‐being. This systematic review aims to summarize the differences in brain structure and function in long‐term practitioners (LTPs) of LKM versus controls to identify possible underlying mechanisms that support mental health and drive treatment effect.

**Methods:**

The literature search included Google Scholar, PubMed, and APA PsycINFO from inception through November 13, 2023.

**Results:**

After review, five studies (64 LTPs and 67 controls total) were included. Brain regions with between‐group differences reported in at least two studies include the superior parietal lobe, inferior frontal gyrus, medial frontal lobe, and insular cortex.

**Conclusion:**

These areas are responsible for self‐compassion, cognitive and affective empathy, and prosociality—personal qualities believed to be fostered through LKM practice. Longitudinal neuroimaging and neurophysiological studies incorporating LKM interventions for specific mental disorders are needed to further inform the biological basis of these treatments and may provide surrogate outcome measures for future clinical trials to refine this promising treatment modality.

## 1 Introduction

Meditation is an ancient practice of “paying attention in a particular way: on purpose, in the present moment, nonjudgmentally” (Kabat‐Zinn et al. 1992). While people have been practicing meditation for centuries, the systematic study of its benefits and mechanisms for improving mental health has only recently begun (Rose et al. 2020; Wang et al. 2021). Meditation practice has been shown to improve emotional intelligence, mental health, and the perception of stressors in comparison to those who do not meditate (Chu 2010). Most literature reports the benefits of specific meditation types, namely mindfulness‐based stress reduction and concentrative meditation (Rose et al. 2020; Wang et al. 2021). However, a growing field of interest has been the exploration of loving‐kindness meditation (LKM), which also provides mental health benefits.

LKM is a specific type of meditation practice that “aims to cultivate unconditional kind attitudes towards oneself and others” (Zeng et al. 2015). Loving‐kindness is one of the “four immeasurable” attitudes in Buddhism, which also include compassion, appreciative joy, and equanimity (Zeng et al. 2017). While loving‐kindness and compassion are considered separate attitudes with their own forms of meditation, LKM practice typically combines various aspects of the Four Immeasurable Meditation styles, including loving‐kindness and compassion meditations, in order to arouse states of loving‐kindness and compassion toward the self and other (Zeng et al. 2017). These states are induced through the repetition of phrases such as “may you be peaceful” (loving‐kindness) or “may you be free of pain” (compassion) while holding an individual in mind (Zeng et al. 2015). LKM is different from more well‐known meditation styles in the United States, such as mindfulness meditation, in which a practitioner “attends to a wide range of changing objects of attention while maintaining moment‐to‐moment awareness,” or transcendental meditation, which a practitioner focuses on a word or phrase repeated during meditation (Kabat‐Zinn et al. 1992).

LKM may provide unique mental health benefits beyond mindfulness by cultivating self‐compassion (Lv et al. 2023), as well as prosocial emotions and behaviors that reduce the physiological stress response (Luberto et al. 2018; Pace et al. 2009; Weinstein and Ryan 2010). Prosocial behaviors, defined as altruistic behaviors with the intention of helping others, are notably beneficial to the individual and to society, associated with better interpersonal relationships, decreased morbidity, and greater physical and psychological well‐being (Luberto et al. 2018). Self‐compassion and mindfulness have been identified as mediators for LKM's benefits in reducing anxiety (Zheng et al. 2023). Meta‐analyses of LKM have found a moderate decrease in self‐reported symptoms of depression and anxiety (Galante et al. 2014; Zheng et al. 2023). Interventions that include LKM have also been developed for posttraumatic stress disorder (Kearney et al. 2021; Lang et al. 2020), borderline personality disorder (BPD) (Feliu‐Soler et al. 2017), eating disorders (Kelly et al. 2017), and depression (Hofmann et al. 2015). Notably, these disorders can be associated with significant self‐criticism and some with a history of interpersonal violence. In this context, it has been shown that exercises that improve self‐compassion are therapeutic by reducing clinical symptoms (Gerdes et al. 2021; Poli et al. 2022).

The mental health benefits of LKM are presumed to be due to neuroplastic changes in the brain that alter gray and white matter morphology and network activation patterns to promote well‐being (Posner et al. 2014; Weng et al. 2013). A meta‐analysis of functional magnetic resonance imaging (fMRI) studies of various meditation styles demonstrated dissociable effects between focused attention practitioners and LKM practitioners, suggesting that different forms of meditation are associated with neuroplastic changes in different brain regions (Fox et al. 2016). In this meta‐analysis, five studies of practitioners engaged in loving‐kindness/compassion meditation demonstrated activation in the right anterior insula/frontal operculum, right parieto‐occipital sulcus, and right somatosensory cortices during meditation. The anterior insula was activated during open monitoring meditation, mantra meditation, and LKM. Further investigation using additional methods other than fMRI was not considered in this study, which showcased a unique opportunity for additional exploration including other modes of brain imaging that our study sought to take advantage of.

The dose effect of LKM on mental health outcomes is unclear but results from short‐term interventions suggest that longer duration of practice produces more significant changes in symptom measures and biological correlates of stress (Zeng et al. 2017). An electroencephalography (EEG) study in long‐term practitioners (LTPs) with up to 40,000 h of experience found a linear relationship between frontal network functional connectivity and hours of meditation (Yordanova et al. 2021). Therefore, we surmise that neuroplastic changes are more likely to be found in the brains of LTPs than novices.

As a neurobiologically based mechanism of action for LKM in improving mental health outcomes is currently lacking, we sought to identify the neural correlates of long‐term LKM practice that may support mental health. To accomplish this, we completed a systematic review as an exploratory search to assess the current state of LKM literature, including multiple types of brain imaging/recording modalities given the presumed limited fund of data from preliminary searches. We pursued this goal with one key question in mind: “What differences in brain structure and function in LTPs of LKM versus controls contribute to improved mental health?”

## 2 Methods

We completed this systematic review in accordance with the Preferred Reporting Items for Systematic Reviews and Meta‐Analyses (PRISMA) guidelines (Moher et al. 2010). Our study protocol was published in PROSPERO (CRD42022324378) before initiating our searches. With the assistance of a medical librarian, we searched Google Scholar, PubMed, and APA PsycINFO from inception through November 13, 2023 (see Supplemental Appendix for search strings). Inclusion criteria were as follows: Studies published in English that included LTPs of exclusively or nearly exclusively LKM (including loving‐kindness and/or compassion meditation) versus a control non‐meditator population, *N* ≥ 10 in both LTP and control groups, and a description of differences in specific brain regions identified using functional or structural magnetic resonance imaging (MRI), EEG, or event‐related potential (ERP). We defined LTPs a priori arbitrarily as individuals with a minimum of 1000 h of meditation experience, as there is no defined criterion for LTP in the meditation research literature. We did this under the notion that measurable differences in structural MRI would require many hours of practice. After conducting our initial searches, a citation search was completed of included studies and relevant systematic reviews. Our review was completed using Covidence systematic review software (Veritas Health Innovation, Melbourne, Australia). Two investigators independently completed title‐abstract screening and full‐text review to determine study eligibility, with differences settled by consensus. We did not initially include positron emission tomography (PET) in our initial search, so we subsequently searched Google Scholar, PubMed, and APA PsycINFO replacing MRI and EEG search terms with PET, without identifying any additional studies at the title‐abstract level.

### 2.1 Extraction

For structural MRI data, we extracted differences in cortical gray matter thickness between LTPs and controls that were statistically significant at a corrected *p*‐value of < 0.05. In the functional MRI studies, we extracted group (LTPs vs. controls) × state (meditative vs. non‐meditative) interactions, and activations during exposure to positive, negative, or neutral stimuli (faces or sounds). For the single P300 ERP study, we extracted areas of significant difference amplitude between LTPs and novices after viewing images of the self versus a close other. Finally, we also extracted scores on self‐report scales, and results of correlation/association analyses between hours of meditation experience and differences in brain structure or function.

### 2.2 Risk of Bias Assessment

A risk of bias assessment was then completed independently by two investigators (KB and SE) using the Newcastle‐Ottawa Scale 2 Case‐Control Scale (NOS) (Wells et al. 2000). Due to the limited number of studies that met inclusion criteria, we could not assess for publication bias.

## 3 Results

**FIGURE 1:**
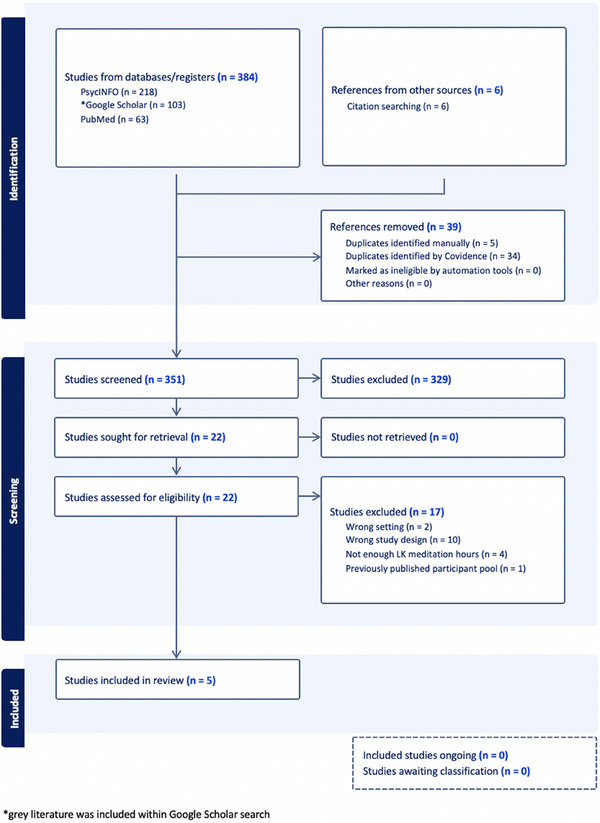
Shows the systematic identification, screening, and inclusion process for determining which journal articles were used for data analysis in this study. A total of 384 studies were identified from database searching and an additional 6 from citation searching. A total of 39 studies were then removed as duplicates, making 351 articles screened by at least two authors. A total of 346 articles were removed for reasons of not meeting inclusion criteria, resulting in 5 articles included for review.

A total of 384 articles were obtained from our database searches (Figure 1). Of these, 329 studies were excluded at the title‐abstract level. These studies were excluded because they did not focus on LKM specifically or they did not involve a study design comparing LTPs of LKM with a non‐meditator population. Twenty‐two studies were reviewed at the full‐text level with 17 excluded (10 for incorrect study design, 4 because the individuals in the LTP group did not meet our a priori threshold for the number of meditation hours, 2 because of the wrong study setting, and one study that reanalyzed previously published data). Some examples of incorrect study design and setting that led to exclusion were the presence of review articles, dissertations, and book chapters as opposed to the desired trial methodology.

We selected five studies for inclusion in our systematic review, with a total of 64 LTPs and 67 control participants. The studies recruited LTPs with a similar duration of meditation experience, largely representing samples of convenience from various meditation centers, meditation networks, or through contact with a well‐respected Buddhist monk. Characteristics of the LTPs in the five studies are listed in Table 1. Of the five studies, two utilized structural MRI to calculate cortical gray matter thickness (Engen et al. 2018; Leung et al. 2013) and two utilized task‐based fMRI studies with either emotionally positive and negative (Lee et al. 2012) or positive, negative, and neutral (Lutz et al. 2008) stimuli. One of the five studies completed both cortical thickness and fMRI analyses (Engen et al. 2018). Finally, one study utilized EEG readings to determine P300 amplitudes in an oddball paradigm including faces of the self and an emotionally close individual, hereby referred to as a close other (Trautwein et al. 2016). A summary of findings from each of the five studies is included in Table 2.

**TABLE 1:** Characteristics of LTPs.

Study	Number of LKM LTPs	Age (years)	Female (%)	Background	Origin	Hours of meditation
Engen et al. (2018)	17	56 ± 5	29	Tibetan Buddhist Adepts	1 Asian, 1 German, 1 British, 14 French	40k ± 12k
Lee et al. (2012)	11	52 ± 11	NR	Buddhist meditation network members in Hong Kong	Chinese	7.5k ± 6.7k
Leung et al. (2013)	10	50 ± 11	0	Buddhist meditation network members in Hong Kong	Chinese	6.5k ± 6.0 k
Lutz et al. (2008)	15	45 ± 13	13	Tibetan Buddhist experts contacted by a particular monk	9 Asian, 7 European	10k − 50k
Trautwein et al. (2016)	11	42 ± 11	54	Local meditation centers in Germany	Not reported	1.2k ± 2.0k

*Note*: The demographic characteristics and LKM experience of the LTPs in each study selected.

Abbreviations: LKM = loving kindness meditation, LTPs = long term practitioners, NR = not reported.

**TABLE 2:** Summary of included studies and neuroimaging findings.

Author	Number of participants per group	State of participants	Modality	Comparison	Summary of findings (corrected *p* < 0.05)
Structural MRI					
Engen et al. (2018)	17 LTPs 15 controls	Non‐meditative	VBM	Cortical thickness differences in LTPs versus controls	Greater cortical thickness in left insular/ventrolateral prefrontal cortex (IFG) in LTPs vs. controls
Leung et al. (2013)	10 LTPs 15 controls	Non‐meditative	VBM	Cortical thickness differences in LTPs vs. controls	Greater cortical thickness in right angular gyrus and right posterior parahippocampal gyrus in LTPs vs. controls
Task‐based functional MRI					
Engen et al. (2018)	17 LTPs 15 controls	Meditative and rest	fMRI during meditation and rest	Areas of activation in LTPs vs. controls during meditation	Left fronto‐polar cortex (LTPs>controls) Left centro‐parietal cortex, left medial occipital (controls>LTPs)
Lee et al. (2012)	11 LTPs 11 controls	Meditative and rest	Task based fMRI viewing emotionally valenced (positive/happy vs. negative/sad) faces	Group × state interaction for valenced stimuli	*Viewing positively valenced (happy) faces*: Left ventral ACC and right IFG (LTPs > controls during meditation) *Viewing negatively valenced (sad) faces*: Left MFG and left caudate (LTPs > controls during meditation) Right caudate (controls > LTPs during rest)
Lutz et al. (2008)	15 LTPs 15 controls	Meditative and rest	Task based fMRI with emotionally valenced (positive/negative/neutral) sounds	Group × state interaction Group × state by valence interaction	Right TPJ, right IFG, mPFC, precuneus/PCC (LTPs > controls in meditation vs. rest when listening to emotional sounds) Right insula, right somatosensory cortex, right superior temporal gyrus (LTPs > controls, emotional > neutral, meditation > rest) Right anterior insula/IFG (LTPs > controls, emotional > neutral)
Structural/functional MRI overlap					
Engen et al. (2018)	17 LTPs 15 controls	Meditative and rest			Left ventrolateral prefrontal region (IFG)
Evoked related potential					
Trautwein et al. (2016)	11 LTPs 11 controls	Rest during presentation of self vs. close other faces	EEG	P300 amplitude in self vs. other	Higher amplitude in self‐other stimuli at Pz in controls vs. no difference in LTPs

*Note*: A summary of significant neuroimaging findings from each study. Findings are organized based upon the modality used (structural MRI, task‐based functional MRI, structural/functional MRI overlap, evoked related potentials). For each finding, the state of the participants during the imaging (meditative vs. non‐meditative), modality used, and comparison drawn are delineated. Specific brain regions are identified as having higher or lower structural or functional capacity in LTPs versus controls.

Abbreviations: ACC = anterior cingulate cortex, AI = anterior insula, EEG = electroencephalography; fMRI = functional magnetic resonance imaging, IFG = inferior frontal gyrus, LKM = loving kindness meditation; LTPs = long term practitioners, MFG = medial frontal gyrus, mPFC = medial prefrontal cortex, PCC = posterior cingulate cortex, VBM = voxel based morphometry.

### 3.1 Cortical Thickness Differences in LTPs Versus Controls

Leung et al. reported that LKM experts had more gray matter in the right angular gyrus and right parahippocampal gyrus in LTPs versus controls (corrected *p* < 0.05) (Leung et al. 2013). Engen et al. found significantly greater cortical thickness of the left insular and ventrolateral cortices in LTPs compared to controls (*p* < 0.029), and a trend toward increased thickness in the right fronto‐polar cortex *(p* = 0.069) (Engen et al. 2018). No areas with less cortical thickness in LTPs versus controls, or interhemispheric asymmetries were identified (Engen et al. 2018).

### 3.2 fMRI Activation Pattern Differences in LTPs Versus Controls

Differences in activation patterns in LTPs versus controls in the meditative state included the following: increased activity in the left fronto‐polar cortex (*p* = 0.001) and decreased activity in the left centro‐parietal and left medial occipital regions (*p* = 0.0001 and *p* = 0.00015, respectively) in one study (Engen et al. 2018), and increased activity in the right temporoparietal junction (TPJ), right IFG, medial prefrontal cortex (mPFC), and precuneus/posterior cingulate cortex (PCC) *(p *< 0.01) in another study (Lutz et al. 2008).

Two studies, Lee et al. (2012) and Lutz et al. (2008), identified differences in activation patterns in LTPs versus controls in the meditative state when additionally exposed to a positive, negative, or neutral stimulus. When meditating, LTPs viewing a positively valenced stimulus (happy faces) demonstrated increased activity in the right inferior frontal gyrus (IFG) and left ventral anterior cingulate cortex (ACC) versus controls (Lee et al. 2012). Greater ACC activation was also present in LTPs viewing happy faces when at rest versus controls (*p *< 0.001) (Lee et al. 2012). When meditating and viewing a negatively valenced stimulus (sad faces), LTPs demonstrated increased activity in the left medial frontal gyrus (MFG) and left caudate when compared to controls, while controls demonstrated greater activity in the right caudate compared to LTPs (Lee et al. 2012). Finally, increased right insular activation with negative versus positive emotional stimuli (sounds) in LTPs versus controls when meditating versus rest was reported (Lutz et al. 2008).

### 3.3 ERP Differences in LTPs Versus Controls

One study compared P300 amplitudes in LTPs versus control in an oddball paradigm and found a significant difference in amplitude at the Pz electrode between images of self versus close other in controls, versus no difference in LTPs (Trautwein et al. 2016). The Pz electrode measured electrical signals in centroparietal and angular gyrus regions (Trautwein et al. 2016).

### 3.4 Summary of Imaging/ERP Findings

When combined, the results of the 5 studies showed the most consistent differences between LTPs and controls in the following regions: large regions of the parietal lobe encompassing the centroparietal cortex/TPJ/angular gyrus/precuneus, IFG, insula, and mPFC/frontopolar cortex (See Table 3).

**TABLE 3:** Anatomical regions most represented among included studies.

Region	Findings	Comparison	Reference
Centroparietal cortex/TPJ/angular gyrus/precuneus	Greater cortical thickness (right)	LTPs vs. controls	Leung et al. (2013)
	Increased activation in meditation (right)	LTPs vs. controls	Lutz et al. (2008)
	Increased activation in meditation (left)	Controls vs. LTPs	Engen et al. (2018)
	Decreased self vs. other P300 amplitude difference	LTPs vs. controls	Trautwein et al. (2016)
Insula	Increased cortical thickness (left)	LTPs vs. controls	Engen et al. (2018)
	Increased activation in meditation (right) (emotional vs. neutral stimuli)	LTPs vs. controls	Lutz et al. (2008)
mPFC/frontopolar	Increased activation in meditation (left)	LTPs vs. controls	Engen et al. (2018)
	Increased activation in meditation	LTPs vs. controls	Lutz et al. (2008)
IFG	Increased cortical thickness (left)	LTPs vs. controls	Engen et al. (2018)
	Increased activation during meditation and viewing happy faces (right)	LTPs vs. controls	Lee et al. (2012)
	Increased activation in meditation and during emotional stimuli (right)	LTPs vs. controls	Lutz et al. (2008)
	Overlap of structural/functional differences (left)	LTPs vs. controls	Engen et al. (2018)

*Note*: The brain regions most represented amongst all significant findings from the five included studies. The specific neuroimaging findings in each study that correlate with the four brain regions identified are included for reference.

### 3.5 Differences in Self‐Report Measures in LTPs Versus Controls

No differences in Beck Depression Inventory (BDI‐2) or Spielberger Trait Anxiety Scale (STAI‐T) scores were found between LTPs and controls in one study (Engen et al. 2018). Greater negative affect was reported in controls versus LTPs on the Chinese Affect Scale in one study (Lee et al. 2012). Another study found LTPs scored higher on the Compassionate Love Scale, Stranger‐Humanity Version (*p* = 0.01), and Self‐Compassion Scale (*p* = 0.03), but not on the Compassionate Love Scale, Specific Close Other Version, or Inclusion of Other in Self Scale (Trautwein et al. 2016).

### 3.6 Correlations Between Hours in LKM and Brain Structural/Functional Measures

Correlations between time spent in LKM and structural or functional measurements were inconsistent, with one study reporting that the cortical thickness of the right angular gyrus correlated with hours of meditative experience, (*p* = 0.028) (Leung et al. 2013), and another that found no association between hours of experience and functional or structural measurements (Engen et al. 2018). Both of these studies corrected for age and years of education in their correlations.

### 3.7 Risk of Bias Assessment

Using NOS criteria, two investigators rated the studies individually as moderate to low risk of bias based on the following criteria: 0–3 = high risk of bias, 4–6 = moderate risk of bias, and 7–9 = low risk of bias. One of the studies (Engen et al. 2018) was rated a moderate risk of bias because of a case definition based on self‐reports, unblinded exposure ascertainment, and a different method of ascertainment for cases and controls. All other studies (Lee et al. 2012; Leung et al. 2013; Lutz et al. 2008; Trautwein et al. 2016) were rated as low risk of bias.

## 4 Discussion

Our literature search yielded five controlled studies of LTPs of loving‐kindness/compassion meditation who completed neuroimaging or ERP assessments. The studies varied in design, particularly the two task‐based functional neuroimaging studies. Despite the design differences, the studies demonstrated an interesting convergence of structural and functional differences in the brains of LTPs as compared to controls in a few relatively specific regions: the superior parietal lobe, IFG, medial frontal lobe, and insular cortex. The following section serves to delve further into these regions while making inferences based on the data to connect brain matter and activity to common mental health conditions that loving‐kindness/compassion medication may help alleviate.

### 4.1 Superior Parietal Lobe

The region of greatest overlap among the studies was the superior parietal lobe, including the TPJ and angular gyrus. LTPs had greater cortical thickness in the right angular gyrus (Leung et al. 2013), and the right TPJ showed greater activation during meditation in LTPs versus control participants (Lutz et al. 2008). Interestingly, the *left* TPJ was more active in control participants versus LTPs during meditation (Engen et al. 2018). These findings suggest neuroplastic changes that produce a left‐to‐right shift in parietal lobe activity during LKM with long‐term practice. In addition, long‐term practice was associated with a decrease in P300 amplitude in the centroparietal region when viewing images of the self versus a close other, suggesting less self‐referential thinking (Trautwein et al. 2016).

These findings have potential implications for improving some symptoms of psychiatric conditions. For example, there is decreased functional connectivity between TPJ and many areas of the brain in major depressive disorder (MDD) and early schizophrenia compared to healthy controls (Penner et al. 2018), and decreased activity in the right angular gyrus was found in non‐medicated patients with schizophrenia compared to controls in a meta‐analysis (Gao et al. 2018). These regions are associated with cognitive empathy (theory of mind) and emotional empathy, which support social functioning and are known to be decreased in MDD and schizophrenia (Liang et al. 2020). This suggests that stimulation of these regions through LKM practice may promote prosocial behavior and improved effect. LKM has demonstrated efficacy in reducing negative affect in those with MDD and lowering self and clinician‐reported depression ratings (Hofmann et al. 2015). In addition, LKM has been shown to reduce negative symptoms and increase the intensity and frequency of positive emotions in schizophrenic individuals (Johnson et al. 2011).

### 4.2 Inferior Frontal Gyrus

The IFG emerged as another region of converging structural and functional differences between LTPs and controls within our review. In LTPs, increased activity was noted during meditation while viewing happy faces in the right IFG relative to controls (Lee et al. 2012). In addition, there was increased cortical thickness of the left IFG in LTPs compared to controls (Engen et al. 2018).

The right IFG has been found to be consistently activated in systematic reviews of functional imaging studies of empathy and compassion (Hou et al. 2017; Novak et al. 2022). In an fMRI meta‐analysis, the IFG is one of several regions (along with periaqueductal gray, anterior insula, and ACC) to be activated during states of compassion (Kim et al. 2020). A reduction in IFG activity may be indicative of a decreased ability to empathize with self and others, which may contribute to negative symptoms in schizophrenia (Shaffer et al. 2015). In a pilot study, LKM has been shown to significantly reduce negative symptoms and increase positive affect in those with schizophrenia (Johnson et al. 2011), possibly due to stimulation in these regions.

### 4.3 Medial Prefrontal Cortex

The studies extracted demonstrate overlapping structural and functional differences between LTPs and controls in the mPFC. For instance, increased activity was noted in the left mPFC during meditation compared to controls (Engen et al. 2018), with another study indicating increased activity in bilateral medial prefrontal cortices during meditation (Lutz et al. 2008).

Clinically, alterations in the function of the medial frontal cortex have been demonstrated in post‐traumatic stress disorder (PTSD), schizophrenia, and generalized anxiety disorder (GAD) (Graser and Stangier 2018). There is decreased activity in the mPFC in GAD and schizophrenia, and both decreased activity and reduced volume in PTSD (Duval et al. 2015; Shaffer et al. 2015). The function of the mPFC is to attend to higher‐level cognitive tasks and regulate thought and action (Graser and Stangier 2018). Specifically, it has been shown that the medial frontal cortex is consistently activated in planning and evaluating the worth of prosocial acts (Bellucci et al. 2020). It is likely that the decrease in activity in these regions indicates a difficulty with emotional regulation and poor socio‐cognitive abilities that negatively influence an individual's ability to engage in prosocial behaviors. LKM could potentially ameliorate the emotional dysregulation and decreased prosocial behavior seen in these psychiatric conditions.

### 4.4 Insular Cortex

One of the last notable regions that shows significant structural and functional differences between LTPs and controls is the insular cortex. Increased activity is present in the right insular cortex during meditation, and there is increased cortical thickness in the left insular cortex in LTPs (Engen et al. 2018; Lutz et al. 2008). Of note, insula activation is not unique to the LKM style and is noted to be present in focused attention, mantra recitation, and open monitoring meditation styles as well (Fox et al. 2016).

GAD, BPD, and schizophrenia are associated with dysfunction in the insular cortex. Worry severity in GAD has been directly correlated with measures of connectivity involving the right anterior insula compared to healthy controls (Andreescu et al. 2015). Schizophrenia has shown decreased activity in the bilateral insula compared to healthy controls (Shaffer et al. 2015), and BPD demonstrates both increased and decreased activity in bilateral anterior insulae after dialectic behavioral therapy with decreased activity in bilateral insulae associated with symptom improvement (Iskric and Barkley‐Levenson 2021). Activation of the insular cortex specifically is indicative of greater experiences of empathy across functional imaging studies, with the right insula primarily associated with affective‐perceptual empathy and the left with both affective‐perceptual and cognitive evaluative empathy (Fan et al. 2011). Past studies have shown that LKM has assisted in reducing self‐criticism and improving self‐kindness in those with BPD, and in reducing anxiety in a combined LKM/compassion meditation practice, potentially through the proposed above mechanism (Feliu‐Soler et al. 2017; Zheng et al. 2023).

### 4.5 Summary of Findings

Taken together, the known overlapping social cognitive functions of the superior parietal lobe, IFG, mPFC, and insula suggest LKMs benefits in reducing symptom burden in clinical populations through increasing self‐compassion and capacity for cognitive and affective empathy that support prosocial behavior and improved effect. However, clinical correlations through self‐report were limited in the five studies that met our inclusion criteria.

## 5 Research Gaps and Areas for Further Study

The possible neural mechanisms for LKM's benefits in improved effect and prosocial behavior are intriguing. Nevertheless, the existing literature on the neuroimaging correlates of long‐term LKM practice with suitable controls is notably limited, as evidenced by the inclusion of only five articles meeting the inclusion criteria in the current review. Before conducting the review search, 1000 h of LKM experience was deemed sufficient to distinguish an LTP from a novice to investigate the dose‐effect relationship of LKM. The existing literature to date simply does not contain much data about meditators that fit this criterion. This criterion was selected to affirm that sufficient structural and functional changes would be clear in data analysis. It is unclear how many minimal hours are needed for structural brain changes. Functional change has been noted in short‐term training in the literature (Mora Álvarez et al. 2023; Bremer et al. 2022), but the minimum training to achieve structural change is less clear. It is possible that less than 1000 h could be needed to see effective structural changes. In addition, the inclusion of terms such as “connectivity” could have resulted in more papers using resting‐state fMRI, which was notably absent from our included studies. Despite this and the limited number of studies, the presence of consistent and complementary findings within this small pool suggests that the findings are relatively robust in LTPs.

This review, in the context of limited data on LKM, was performed with the intention of providing an investigative look into the current data that exists regarding LKM's effect on neuroplasticity as it relates to improved mental health. Given the small fund of papers that met inclusion criteria, some heterogeneity was introduced through the inclusion of multiple different imaging studies, neuroimaging statistical techniques, and LTPs with experience in forms of meditation other than LKM. This heterogeneity was impossible to avoid given the lack of existing data, highlighting the importance of additional research that can increase the available funds for data and further support this review's findings. Specifically, an increase of LTPs with sole experience in LKM would help disentangle results from the potential influence of previous practice of other meditation types. In addition, our discussion connecting the areas of the brain identified in the papers with mental health pathophysiology remains limited, as it is based upon inference from the existing literature due to a lack of data directly investigating brain structure and function in LKM practitioners with mental health disorder progression. Given the current state of published data on this subject, these inferences were necessary and unavoidable in order to theorize the demonstrated connection between LKM practice and improved mental health. More direct literature is needed to consolidate the inferences presented here.

Another limitation of the current literature is the cross‐sectional nature of the studies. While neuroplasticity is presumed to be the mechanism that drives the differences in brain structure and function by the authors of the studies reviewed, longitudinal studies are needed to support this claim. It is possible that the LTPs who participated in these studies were more prosocial and biased toward kindness/compassion before choosing to practice LKM. However, given the costs associated with longitudinal imaging studies and the necessary expertise, it is possible that future controlled studies of the long‐term neuroimaging correlates of LKM interventions will be rare.

An important consideration in the LTP group is that the practitioners may be socially engaged or practicing loving‐kindness or compassion as part of their daily life, outside of meditation practice. LKM has previously been seen to support prosocial behavior, and many of the convergent regions we identified are activated during interpersonal interactions. Therefore, the changes we report may be a function of a way of life, rather than simply a meditation practice.

## 6 Conclusion

In summary, the limited published literature investigating neuroimaging and neurophysiological changes in LTPs of LKM suggest neuroplastic changes in four key brain regions associated with clinical symptoms of multiple psychiatric illnesses. Adjunctive LKM‐based interventions show promise in limiting suffering and improving well‐being and prosocial behaviors in diverse disorders such as MDD, schizophrenia, BPD, and PTSD. Studies investigating the neural correlates of LKM treatment response in clinical populations are needed to bridge the gap in understanding how this promising intervention leads to clinical changes. Additionally, a greater understanding of functional and structural imaging correlates of treatment response may offer surrogate outcome measures in future clinical trials.

## Author Contributions

**Kiren Bashir**: conceptualization, writing–original draft, writing–review and editing, investigation, formal analysis. **Stephen B. Edstrom**: conceptualization, data curation, formal analysis, writing–review and editing, writing–original draft, methodology, project administration, investigation. **Sara J. Barlow**: investigation, formal analysis, writing–review and editing, writing–original draft. **Danielle Gainer**: writing–review and editing, software, resources. **Jeffrey D. Lewis**: supervision, investigation, methodology, writing–original draft, writing–review and editing, formal analysis.

## Disclosure

The views presented in the paper represent the opinion of the authors, and not the United States Air Force or Department of Defense.

## Ethics Statement

The systematic review was conducted in accordance with Preferred Reporting Items for Systematic Reviews and Meta‐Analyses (PRISMA) guidelines.

## Conflicts of Interest

The authors declare no conflicts of interest.

### Peer Review

The peer review history for this article is available at https://publons.com/publon/10.1002/brb3.70372.

## Supporting information



Supporting information

## Data Availability

The data supporting the findings of this study are available upon reasonable request from the corresponding author.
