# Supplementary material for: Loving‐Kindness Meditation: Systematic Review of Neuroimaging Correlates in Long‐Term Practitioners and Clinical Implications
Source: Brain Behav. 2025 Feb 28;15(3):e70372. doi: 10.1002/brb3.70372 (PMC11870839; doi:10.1002/brb3.70372)
Supplement: Supplementary file 1 — Supporting information [file BRB3-15-e70372-s001.docx]

Supplemental Appendix

I. Search Strings

Google Scholar

metta compassion loving kindness "meditation" MRI plasticity EEG.

PsycINFO

(KW metta meditation OR loving kindness meditation OR compassion meditation AND (KW mri or magnetic resonance imaging or eeg or electroencephalogram or electroencephalography or fmri or functional magnetic resonance imaging or plasticity or neuroplasticity)

hit 218

PubMed

("metta"[All Fields] OR (("love"[MeSH Terms] OR "love"[All Fields] OR "loved"[All Fields] OR "loves"[All Fields] OR "loving"[All Fields] OR "love s"[All Fields]) AND "kindness"[All Fields] AND ("meditate"[All Fields] OR "meditated"[All Fields] OR "meditating"[All Fields] OR "meditation"[MeSH Terms] OR "meditation"[All Fields] OR "meditations"[All Fields] OR "meditation s"[All Fields] OR "meditational"[All Fields] OR "meditative"[All Fields] OR "meditator"[All Fields] OR "meditators"[All Fields])) OR (("empathy"[MeSH Terms] OR "empathy"[All Fields] OR "compassion"[All Fields]) AND ("meditate"[All Fields] OR "meditated"[All Fields] OR "meditating"[All Fields] OR "meditation"[MeSH Terms] OR "meditation"[All Fields] OR "meditations"[All Fields] OR "meditation s"[All Fields] OR "meditational"[All Fields] OR "meditative"[All Fields] OR "meditator"[All Fields] OR "meditators"[All Fields]))) AND ("neuronal plasticity"[MeSH Terms] OR ("neuronal"[All Fields] AND "plasticity"[All Fields]) OR "neuronal plasticity"[All Fields] OR "neuroplasticity"[All Fields] OR "neuroplastic"[All Fields] OR ("electroencephalography"[MeSH Terms] OR "electroencephalography"[All Fields] OR "eeg"[All Fields]) OR ("magnetic resonance imaging"[MeSH Terms] OR ("magnetic"[All Fields] AND "resonance"[All Fields] AND "imaging"[All Fields]) OR "magnetic resonance imaging"[All Fields] OR "mri"[All Fields]) OR ("magnetic resonance imaging"[MeSH Terms] OR ("magnetic"[All Fields] AND "resonance"[All Fields] AND "imaging"[All Fields]) OR "magnetic resonance imaging"[All Fields] OR "fmri"[All Fields]))

Hit 63
